# A Letter from Dr. Mercer, U. S. N.

**Published:** 1838-10-10

**Authors:** John C. Mercer

**Affiliations:** Passed Assistant Surgeon of the U. S. N.; U. S. Naval Hospital; Near Norfolk, Virginia


### A LETTER FROM DR. MERCER, U. S. N.

U. S. Naval Hospital, *Near Norfolk, Virginia*, Sept. 24th, 1838.

To the Editors of the Medical Examiner.

*Gentlemen:*—If you have room for a few desultory observations, in your very interesting and punctual periodical, I will ask the favour of a corner of it to invite the attention of your readers to a point in Therapeutic Medicine, of such practical importance and such common prevalence, that it is astonishing medical writers have not laid it down in more emphatic language. I allude to the connection that exists between pains in the extremities, especially the lower, and diseases of the digestive apparatus. Let me be understood: I refer, not to that costive condition of the bowels, in which there is a mechanical cause for such symptoms, but to gastric, hepatic, and enteritic affections, distinguished for a very opposite state of the intestinal canal. In slight dyspeptic cases this erratic neuralgia very often occurs, as I have often noticed in my own person. Now, every one will at once acknowledge, that it is of primary consequence to discriminate between these pains and those of rheumatic irritation, since the remedies appropriate in the one are counter-indicated in the other. Yet this error in diagnosis is very often committed, as I have had occasion after occasion tc observe, and the remembrance of an instance, in which I was guilty of such misapprehension, will ever remain fresh in my recollection. To avoid all misconception, in such cases, it is merely necessary to employ that strict scrutiny which should characterize our examination of a patient's symptoms.

Seeing no direct cause of sympathy between parts of the system, so utterly disconnected in functions, and so remote in situation, I was, at first, wont to ascribe to the imagination of the sufferer, what a little experience has shown not to be ideal. In cholera morbus we have a very forcible exemplification of the point under consideration. Here there are most violent pains and spasms in nearly all the voluntary muscles, and, perhaps, in the excessive vomiting and purging, we have evidence of these phenomena extending to the muscular coats of the stomach and bowels. At least, there is indisputable proof of much gastric and intestinal derangement, looking to the mucous membrane alone. Would it be carrying the matter too far to consider the simultaneous existence of the milder abdominal affections and these erratic pains of the extremities, as cholera in miniature?

I, by no means, gentlemen, lay claim to any new discovery. My object, in making these remarks, is to awaken investigation on this subject, and to give to it all the importance my feeble efforts may impart. Young practitioners will, in a moment, perceive how essential it is to the well-being of their patients, that they neglect the seat for the source of this affection of the extremities, and make their prescriptions conform to the nature of the internal malady.

How are we to explain this conjunction of facts? Shall we say that the spinal nerves, communicating with the seat of disease, convey the morbid influence to the point of their origin, whence it is transmitted through the other cords of the medulla, or that the irritation is radiated through the branches of the trisplanchnic, said to embrace or pursue the course of the arteries. In candour, let it be remarked, this is all conjecture. The fact we know; the rationale of it is yet among the many mysteries of man's structure.

The remarks of Dr. Meigs in one of your late numbers, in relation to Hope's Nitrous Acid Mixture, find ample confirmation in the practice of this hospital.

I am, gentlemen,

Your obedient servant,

John C. Mercer.

*Passed Assistant Surgeon of the U. S. N.*

[Dr. Mercer's position in the naval hospital at Norfolk, gives particular interest to the results of his experience in the treatment of dysentery. We take the opportunity to ask from him a detailed account of his observations in this affection, and in the tropical fevers engendered upon our southern coast and the West India naval station, which his ample materials, from the cases of the sailors who enter the Norfolk hospital, will render exceedingly valuable.—Eds.]

